# "China" as an Analytical Concept: A New Beginning for Chinese Political Science

**DOI:** 10.1007/s41111-021-00195-0

**Published:** 2021-10-19

**Authors:** Xiangmin Wang

**Affiliations:** Political Science, East China Normal University, Shanghai, 200062 China

**Keywords:** China, Chinese political science, Comparative political science, History of political science, Internal shift

## Abstract

The emergence in recent years of a large number of institutional concepts in the world of Chinese political science indicates that Chinese political science is experiencing an "internal shift" that is different from the complete Westernization of the past. Chinese political scientists are seeking theoretical explanations for China's political development based on China's internal context and are looking to provide intellectual arguments for China's modern state building. In this paper, it is proposed that the core of this internal shift of Chinese political science is the consciousness of "China" as an analytical concept, and that China is not only an object of description, but also an analytical perspective for explaining "what is China". Such a view is different from that held by the European and American left and pure traditional researchers or reactionists. On the one hand, this paradigm provides more universal political knowledge in the sense of comparative political science; on the other hand, it can advance Chinese political research by drawing a clearer and more accurate knowledge map of Chinese politics. The emergence of institutional concepts in Chinese political science implies that Chinese political science as a discipline is increasingly moving from the "form" of discipline establishment to the "content" of "what is China". This signifies a real new beginning.

This paper provides a commentary on a series of institutional concepts of Chinese political science in recent years and presents some observations on trends in Chinese political science. The author believes that Chinese political science has experienced an “internal shift” away from the West, which can be seen as a political answer to the question of “what is China”. What stands at the core is the methodological significance of “China” as an analytical concept. The “internal shift” signals a new beginning in Chinese political science.

The interaction between China and the West in modern times facilitated the entry of political science into China, and it was established as a foreign discipline. The changes brought about by the interactions shaped the discipline in China and its knowledge realm has strong extraterritorial attributes. The inconsistency between the established discipline and the traditional Chinese knowledge system indicates that the development of the discipline in modern times was almost a copy of the Western practice. This is in line with the basic law of embryology. Take the conceptual system in political science research as an example. Almost all the concepts used in Chinese political science come from Europe and the United States, and a few local Chinese concepts have been included in the family of political concepts. Two dimensions determine which concept can be treated as a local concept of comparative political science^1^: First, it is a description of local political phenomena, and second, this descriptive concept performs an analytical function. An analytical concept refers to a concept that can give a comprehensive explanation by summarizing or extracting the core variables of phenomena and explaining their causal mechanism using classification and comparison.

In recent years, the manner in which new concepts have been added into Chinese political science has changed, with the emergence of a large number of institutional concepts based on China. These institutional concepts are descriptions of local Chinese political phenomena, and what is more important is the development of a series of analytical institutional concepts. Analytical institutional concepts, which transcend empirical phenomena, describe and explain the state of China, the current Chinese political power structure and its operation, and the social basis of Chinese politics. They also include reflection on and application of the “methodology” in Chinese political research. These analytical organizational concepts are not slogans or sparsely scattered individual research results. Rather, they have created a holistic landscape, using “China” as an analytical concept to provide explanatory political knowledge about “What is China”. Such a holistic landscape and its climate enable us to assert that Chinese political science has experienced an “internal shift” to get rid of the earlier total Westernization.

This paper consists of two parts. The first part begins with the institutional concepts of Chinese political science in recent years and categorizes and integrates them into the spectrum of political issues as a “community building”. The second part summarizes the development trends of institutional concepts in recent years as an internal shift of Chinese political science, and provides a commentary on its meaning and significance from the perspective of the discipline’s history. The key argument is that Chinese political science, after the development of “form” in modern times, is moving toward the “content” within the internal context of China, and that “China” has become an analytical concept in Chinese political science. Chinese political science has a new beginning.

## Institutional Concepts and Pedigree in Chinese Political Science

All communities have their own structures, which evolve and become fixed in the course of history and experience. The fact also applies to empirical research on Chinese political science. It is possible to overlook the form of Chinese political science from a structural point of view, and to understand its formation process from the course of history and experience. We have generalized and summarized the institutional concepts in Chinese political science in recent years by incorporating them into the structure of “community building”, hence the knowledge pedigree of Chinese political science. “Community building” refers to the structure, growth, and formation of an independent regional community.

### How Did China Come into Being, How Has it Survived, and How Has it Been Governed?

Chinese cultural studies have always believed that Chinese culture has its own uniqueness. Japanese scholar Yuzo Mizoguchi also proposed a “Chinese basal body theory”. After these theories and concepts were questioned in the twentieth century, have returned to the minds of Chinese political scholars in recent years and prompted the establishment of a series of institutional concepts.

1.The nature of the community or the relationship between politics, culture, and education. Liang Shuming argues that Chinese culture is a “precocious culture”, and precocity determines the isomorphic relationship between politics, culture, and education in the Chinese community. This constitutes a legitimate argument toward community stickiness, thereby freeing China from the pure power violence and allowing it to become a “civilized state”. Zhao Dingxin observes that imperial China was different from other civilizations, especially Western civilization, in at least seven aspects politically (Zhao 2006). He attributes them to the unified bureaucratic empire driven by wars during the Spring and Autumn Period and the Warring States Period. During the 80 years after the Qin Dynasty, this political process was crystallized into the Confucian-legalist state under Emperor Wu of the Han Dynasty. In the unified bureaucratic empire, political power and ideological power are unified, military power restricted, and economic power marginalized (Zhao 2006). Zhao reasons that the Confucian-legalist state served as the basic form of Chinese politics for more than 2000 years as there is an interdependent symbiotic relationship between the state’s political power and ideological power (Confucianism), with the Confucian class at the hub of the “politics–culture–education” structure. “Politics–culture–education” points to power, ideology, and assimilation. The traditional China dominated by Confucianism was a “cultural and educational state” (Yao 2013a, 2014), and the cultivation of humanities lies in learning culture rather than relying on divine enlightenment. As promoted by the Confucian group, politics and education are interdependent, “combined and separated”, which gives Chinese administration its political quality and gives politics a Confucian idealistic color. The state and society are both placed in the Confucian “community of knowledge and ethics”, and this leads to cooperation rather than resistance between them (Yao 2014).

2.The continuation of the unification history of the community. Methodologically, Yuzo Mizoguchi’s “Chinese basal body theory” expresses the “variation” and “constancy” of empirical positivism, that is, no matter what changes, some factors always recur repetitively or cyclically. These constant factors constitute the structure or structural regulations of the community. The Chinese political community has always been maintained through unification. The officially revised *Twenty-Four Histories* shows that the regime of each dynasty (collective memory of community values) regards itself as the heir of the unification of the previous dynasty, and Chinese political history is a history of dynasty change under unification. However, this common sense has been challenged by Western historians in recent years. Studies, such as the “New Qing History” and the “Inner Asia Theory”, argue that instead of a continuation of traditional dynasties, the Manchu dynasty established an alien regime after entering the Central Plains. Therefore, traditional China was not a unified community. Japan’s Kodansha titled the Song dynasty as the “Era of Nations” of the Liao, Jin, and Song dynasties (Sugiyama 2014), and vigorously propagated the “Mongol Empire” (Sugiyama 2015).

Yang Nianqun's stand on the “Great Unification” is to refute the “New Qing History” and the “Inner Asia Theory”. Yang cites the three elements of great unification from *Chunqiu Gongyang Biography* in the pre-Qin period: The maximum territory in space, the “rightness” and “crookedness” to maintain the rule (the inheritance of the mandate of heaven and the cycle of dynasties), and the “virtue” of gaining support through comforting the people. A difference is that during the Song and Ming dynasties, forced by the northern minorities of Liao, Xixia, Jin, and the later Yuan dynasty, the Song court began “repelling the barbarians”, meaning that the nomads were not eligible for the Central Plains regime. Yang focuses on the Qing history during its early and middle periods, and notes that emperors like Kangxi and Qianlong established the “Imperial Classics” by compiling and reviewing books, such as *Chunqiu* and *ZizhiTongjian* (including the *Second* and *Third Revisions*). Emperor Yongzheng personally examined the case of Zeng Jing and compiled his argument into the widely communicated *Da Yi Jue Mi Lu*, Tibetan Buddhism was legitimized, and there were moral reasons for the war against Xinjiang. These facts clearly explain how the Manchu, as a nomadic nation, consciously merged into the Central Plains, resumed the “mandate of heaven” under the regime and became “orthodox”. The naturalization of the Qing emperors and the “Imperial Classics” were also recognized and promoted by folk scholars, and became the popular concept and collective action of the Qing court and folk scholars (Yang 2018, 2020a, b).

Yang’s research on the "Great Unification" of the Qing dynasty mainly focuses on demonstrating the "orthodox" nature of the dynasty. Yet, there is a modern dimension in the "Great Unification" of the Qing dynasty, which came to dominate the Central Plains as a minority nationality. That is the shaping and formation of a multi-ethnic country or the Chinese nation (Yang 2018b). There is a fundamental difference between nation-states that originated from European absolutist countries and traditional multi-ethnic communities, with respect to the formation of nations. Zhou Ping’s "nation studies" (Zhou, 2021a, b, c) and Huntington’s question of "Who are we" all point to the challenge posed by immigrants and marginalized groups to the assumption of a traditional single nation-state in the modern context. The "nation" of China is not purely about the identity of marginalized groups brought about by modernity in recent years. It is more about the fragile architecture of the "Chinese nation" brought about by regional ethnic autonomy and the right of self-determination for the ethnic minorities behind it, which were absorbed in the "national identity" after the founding of the People’s Republic of China (PRC). The identity building of the Chinese nation has become one of the hot topics in political science in recent years. However, to be fair, although the proposition is fundamentally a political one, political science has made fewer theoretical contributions than the history of concepts (Ge 2011; Huang 2017) and ethnology (Fei 1999; Ma 2016). The Chinese nation has its historical origins, yet it is essentially a modern product. Therefore, from the perspective of institutional concepts, there has not been a definite concept about it in political science, but the issue has been adequately addressed.

3.The organizational form of meritocracy adopted by the imperial court. "Meritocracy" is the word used by Professor Daniel Bell to describe the organizational form of the Chinese government (Bell 2016). In fact, the description is not novel. At the beginning of the twentieth century, when Western political science’s doctrine of political system was comprehensively introduced, the system adopted by the traditional China was defined as "meritocracy", and compared with monarchy and aristocracy adopted by the ancient Greece. Meritocracy was criticized not for the values it advocates, but for its operating experience. In other words, meritocracy cannot guarantee that the rulers or bureaucrats of the regime are all capable people. Besides, meritocracy cannot correct itself. Therefore, as the "less poor political system", democracy leads other political systems. Bell's "meritocracy" can provide an obvious explanation for China's political growth since the reform and opening-up.

4.Exposition of political knowledge centering on "governance". The construction of modern Western states revolves around legal power, and their political science is based on the analysis of political systems or institutions. The truth can be found in two kinds and six types of polity proposed by Plato and Aristotle, the political system revolutions before and after the bourgeois revolution, and even the discussion on the form of government in modern political science in the twentieth century. Traditional China has rich experience in legal power (the political views of Confucianism, legalism, and Taoism, the stable court structure, the sound civil service system, and the imperial examination system, which combines political socialization and the selection of officials) and administrative governance. But despite this, traditional Chinese political studies never focused solely on legal power. The fact is that traditional Chinese political theories in historical discourses are more macro and multi-faceted. The inner shift of Chinese political science lies in the move from the traditional analysis thought of as "sagely within and kingly without" to the "theory of governance". The theory of governance shows a clear political interest: it aims "to understand how the fundamental elements of a political system are organized and what kind of integrated relationship is created" (Ren 2019a). In other words, it focuses on the order structure of the political community, with the system or logic as the standard, and combines the concepts, views, and issues in the traditional Chinese knowledge system to form an order theory category. Ren Feng wrote a 600,000-character treatise that provides a detailed description of the theory of governance in historical texts (Ren 2019b) and outlined a four-phase development process from the pre-Qin period to the late Qing dynasty. The core of the theory of governance is "governance" (the maintenance or governance of a community). This includes the governance of doctrine (basic order law and principles), the governance of law (systemic rules and institutions), and the governance of man (the virtue, intellect, and skills of political subjects) at the abstract and conceptual level. The three (whereas, modern Western political science concentrates on the political system) should be combined, not separated. The resultant force of the three creates different theories and historical states. Centering on "governance" obviously fits the pragmatic political science better. "Governance" is not purely power coercion and administrative technology. Instead, it is the theory of wholeness in which the legality discourse, system design, and operating subject are consistent. The theory of "governance" provides a more macro and complete theory of political community maintenance than polity science does. The failure of the reform of political systems in backward countries simply indicates the absence of the theory of whole governance.

### The Power Form of Modern China

The initial phase of a modern state features the reconstruction of power. In Europe, the feudal system with weakened power was transformed into an absolutist state with a centralized monarchy, while in China, the state power was transferred from the hands of the emperor and bureaucrats to the hands of a new political organization, the political party, and the central axis of power was reorganized institutionally in the national realm. The reorganization of the power structure of modern China includes three aspects: one, the centralized or decentralized power structure, and the state capacity or government effectiveness derived from it; two, response to the representative demands of modern civil rights or the republican political system; three, the organizational form of contemporary Chinese political community. The first two concern the government and political principles of comparative political science, while the last one concerns the specific power form of contemporary China.

1.The centralized (political centralization and unification) structure of modern China. Research on this topic was based on Huntington’s “Political Order in Changing Societies” and included in the historical process of China’s modern transformation (Chen 2000, 2001). Yet, an internal explanation has been sought in recent years from the perspective of the great unification of traditional China. There are two directions in the study of the great unification: historiography, especially Yang Nianqun’s research on the Qing history, which focuses on the relationship between the Manchu and the Han and foreign nationalities, highlighting the history of dynasty replacement in traditional China (and it is considered by political scientists as an extension of research on the Chinese nation); political science focuses on the centralized power structure within the country (between government departments and between the central and local governments). For example, Zhou Guanghui notes that relief policies in traditional China helps shape the resilience of a unified country (Zhou 2021a, b, c). Lin Shangli wrote the following words with great enthusiasm: “Great unification is the axis of China. Without great unification, China would lose the foundation and value of its overall survival” (Lin 2017). The centralized structure of great unification is the “constant” throughout the changes of ancient and modern times: “China’s prefecture-county system produced the centralized system of great unification, which, after going through the interaction of heredity and mutation and repeated selection, has become a stable tradition and exerted a profound impact on later generations of Chinese people in terms of their political behavior and choice of institution. The revolutions in modern China have renewed the political meaning of great unification with the slogan of people’s sovereignty. The Communist Party of China (CPC) has rebuilt China within the framework of great unification and regarded people’s sovereignty as the cornerstone of a modern state and a representative government. The principle of centralization and unification is continuation of the tradition of centralized power in a unified country.” (Chen 2021). It can be said that Huntington’s view of centralized political order comes from the political observation of changing societies in transitional countries, and the understanding of the centralized power structure in Chinese political science in the 1990s comes from the historical experience of modern China. It would not be wrong then to say that the internal shift in Chinese political science in recent years can trace its origin to the great unification system in traditional China, while expanding the historical pedigree of the Chinese power structure and making great unification and centralized power the central axis of China’s political power structure.

2.Response to the representative demands of modern civil rights or the Republican political system. The biggest difference between ancient and modern Chinese politics is that modern Chinese politics emphasizes individual civil rights and the representative institutional arrangements, which are what a democracy or republic require of modern China. The study of civil rights and the political systems in modern Western politics focuses on two dimensions: one, since the Age of Enlightenment, civil rights (“natural rights”) have been regarded as the source of the power of the state community (the Social Contract), and their representativeness and their reflection in the national system is discussed (government with limited power and election as a method to make it happen); two, in terms of the form of the state/government system, the discussion is at the level of the type of government, and the source and restrictions of state power are both presented in the polity theory. Chinese political science sees the aforementioned requirement as a consensus and explores and uncovers the Chinese experience within the scope of the “republic”. Thus it has formed institutional concepts that center on people’s democracy and cover concrete forms of representative theories like democratic centralism, the mass line, political consultation, and the united front, and integrating the theory of political system into political analysis. In this respect, the contribution of the political science community is that these concepts, which were used largely in political documents and as political slogans, have been rigorously defined and explained academically.

However, unlike the previous one-way arguments that universalized Western theories, the approach of contemporary Chinese political analysis contains two dimensions: one is about comparison—Western political theories are examined using the theoretical resources of comparative political science and Marx’s critical theory and are applied to the meta-problem; two, starting from this meta-problem, efforts are made to explore the theoretical significance of the Chinese experience and construct the “republic” theory of Chinese politics. For example, there are specific tiers in Chen Mingming's discussion on democracy and representation in the “principles of the Chinese government”: first, as the basis of the legitimacy of modern governance, democracy cannot be ignored; second, the original intention of democracy is majority rule, which is essentially rule by the people; third, European and American democracy does not subvert majority rule, but it denies the directness and reality of rule by the people, turning the majority rule into “multiple majority rules” (Robert Dahl); finally, in China, rule by the people is usually expressed as “the people are the masters of the country”. “The saying that the people are the masters of the country recognizes and respects the value of people’s holism in terms of empowerment. It does not exclude or blindly believe in equal and universal elections in terms of power construction. It emphasizes response, accountability, and joint participation of consociationalism, which features the consistency between the state and society, in the exercise of power. It resorts to mechanisms established in the name of the people, such as judicial relief, administrative arbitration, and autonomous mediation for the protection of rights. It relies on the people’s congress organs at all levels and the supervision of the masses for power control”. “The people are the masters of the country” is not an abstract notion, it has been tested by an array of policies and governance performance. The representation system is embodied in two dimensions: on the one hand, it is similar to the power construction system for organizing and running the government in the representative system, yet “the status and function of the political party is broader than the meaning of a party group in the representative system, that is, the party is not a ‘partial’ organization of a certain class, group, or a wing of society, but a ‘whole’ representative of the fundamental interests of the entire people”. On the other hand, the people’s democratic system integrates the fundamental mechanism of the state responding to society and unifying the people, and this includes specific forms, such as democratic centralism, the mass line, political consultation, and consensus democracy. Therefore, “while the principles of modern Western government include three parts: the representative system, decentralization, and limited government, the principles of contemporary Chinese government can be summarized as: the representation system, centralization, and effective government” (Chen 2018, 2019, 2021). Obviously, this comparative argument suggests quite clearly that there are both similarities and differences between the Chinese government and its Western counterparts.

3.The organizer or institutional form of the contemporary Chinese political community. The core of this analysis is the status of the CPC in Chinese politics and the political form produced by that status. The greatest experience of modern Chinese politics is that the CPC stands at the core of the leadership of the PRC and represents the central axis of the contemporary Chinese political system. That is the starting point of modern Chinese political analysis, whether it is from the perspective of empirical logicism or historical process. On this dimension, there are two propositions: one, research on the “party-state” and party type from the perspective of comparative political science uses the approach of “bringing in the party” (Jing 2019); the CPC becomes an analytical concept of Chinese politics (Jing et al. 2016); and “party-centrism” (Yang 2010) is the theoretical result of both state-centrism and social-centrism; two, the advent of “party–state” and “party-centrism” in the Chinese experience has developed institutional analytical concepts, such as “party–state system”, “party-ruling state”, and “party government system”. The “party–state” structure began in the period of the Nationalist Government, but it did not acquire a mature political form until the founding of the PRC. The analytical concepts have also developed from “party–state system”, “party-ruling state”, and “party–state” to the more academic “party government system”. In Jing’s view, “party government system” has a strong analytical connotation: “As a compound, it surpasses the logic of party organization and the logic of government organization. It has uniquely integrated the two and created a new logic of its own.” More importantly, the “party government system” ensures the analysis of academic norms rather than political documents in an objective and neutral manner, without the value burden of existing vocabulary. It helps give play to the academic skills of description and analysis and build a holistic analysis framework for understanding Chinese politics. (Jing et al. 2016).

### State Governance: The Degree of National Capability or Effectiveness

The idea of “political society” proposed in recent years has greatly broken through the “state-society” theory in the West. It suggests that the development of the West in modern times is not about mutual empowerment through the checks and balances of the state and society, but about the integration and dominance of society by the state through political means (Wang 2017, 2018), hence the state as a Leviathan. The theoretical significance of “political society” is that national capability and governance effectiveness are not exclusive issues for late-developing countries in transition. They are the common experience and proposition for all communities in the building phase.

The history of Chinese research on national capabilities is very clear. Wang Shaoguang and Hu Angang first used the concept of “national capability” and defined it as one of fiscal extraction (Wang and Hu 1993). The concept of “governance” has gone through a shift in China. In the late 1990s, especially at the beginning of the twenty-first century, the Western concept of “governance” was introduced to China, with a focus on “good governance”. Devaluing government functions and boosting the market and society’s ability to supply public products, became a popular theory in China’s academic world. However, in 2013, it was “decided” at the Third Plenary Session of the 18th Central Committee that “the overall goal of deepening reform across the board is to improve and develop the socialist system with Chinese characteristics, and to advance the modernization of the national governance system and governance capability”. “National governance” became a keyword of China’s political reform and soon became a major research field in Chinese political science. The key difference between “national governance” and “governance” is that the former stresses the dominant position of the state and the government in governance. The theory of “governance” is essentially a de-national or de-political market and social supply of public products, whereas “national governance” emphasizes that the state and the government are in the leading position to coordinate the market and society and jointly meet the people’s livelihood needs. Compared with institutional determinism in Western political science, national governance shows dialectical unity of institution and capability by emphasizing both the governance institution (system) and the governance capability. Therefore, some scholars believe that it constitutes a new research paradigm that breaks through the theories of Western countries. The core of national governance is state-led public policy, then research on the policy process in the field of people’s livelihood, especially on the government’s purchase of public services from social organizations. This is one of the central topics in the research on the modernization of the national governance system and governance capability in recent years. (Wang et al. 2012).

It should be noted that research on national capability and governance effectiveness from the perspective of national analysis has already become a major area in political science. Seeing the political decline of European and American countries in the twenty-first century, Fukuyama came up with concepts like “responsible government”. However, the internal shift in Chinese political science considers “national governance” as an alternative paradigm for the “polity theory” in Western political science, arguing that the new agenda of comparative political science should shift from Western-dominated research on institutions to a comparison of governance capabilities (Yang 2020a, b, c). Under this context, the COVID-19 pandemic in 2020 can serve as an unusual case of comparative political science. However, no one has ever been able to explain how “national governance” has become an analytical concept and helped construct a set of national explanatory theories and how Western politics has returned to the theme of state construction. The framework of “governance state” has been set up, but more work needs to be done to elaborate it. That is also the weakest part in the internal shift in Chinese political science.

### The Social Foundation of Chinese Politics

China’s history of over 5000 years has seen numerous wars and frequent replacement of dynasties, long and short-lived. Why can it continue for thousands of years without disruption? Viewing the “change” and “constancy” of traditional China in that context, one can find that the imperial state kept changing, while society was like a reservoir, bringing forth the imperial state. Now, what is the social foundation of traditional China as a reservoir? Is there a new social foundation for today’s Chinese politics?

Unlike the history of thought, which focuses on the Confucian group, political sociology focuses on social organization and social structure and their influence on politics, so it seeks theoretical explanations from field empirical research. It is generally believed that the social foundation of traditional China is blood-related organizations. Before the Song dynasty, it was a patriarchal society, and after the Song dynasty, it was transformed into a lineage society. Both were bonded by blood. However, the clans in the patriarchal society were large lineages and governed by the feudal system in politics. The clans rebuilt in the Song dynasty could only trace back to three or five generations, and the institutional arrangement in politics changed as the feudal system of land-holding was replaced by the prefecture-county-based bureaucratic system. Recent studies have put traditional views in the realm of political science. The theory of “rights from ancestors” incorporates the elements in the political field—life, property, rules; age, gender, identity; location, power, and responsibility—into the rationality and basis of the existence and behavior of people derived from blood lineage. Unlike the binary opposition between society and the state embedded in “natural rights”, what lies in the idea of “rights from ancestors” is the co-existence and co-prosperity between society and the state, which shapes the notion of a community with a shared future for all (Xu 2018). *A Survey of China’s Rural Areas*, the serial field research results of Central China Normal University, demonstrates that the disintegration of clan communities does not mean the disappearance of blood lineage, and that the social organization of “rights from ancestors” is still applicable today.

The geographical expansion of “rights from ancestors” has resulted in the “superposition of (blood-territory) relations”. The “superposition of relations” has produced a “state in relations” and the “household system”, linking society and the state. Xu Yong examines the propositions in the “territory-blood relationship” and argues that traditional China formed a super large territorial state based on the “superposition of relations” (Xu 2020a, b). In traditional monarchy countries, the social organization that connects the family and the state is the “household system”. This combines the basic unit of society that is formed by blood lineage and the basic unit of a territorial state. *BianhuQimin*, a household registration system, changes the way of state governance based on the relationship between individuals, clans, and tribes. In a certain territory, all people are subjects of the state, irrespective of the differences in identity and status caused by the old blood lineage or the differences caused by the new property relations, and all enjoy the public rights and perform obligations granted by the state according to their place of residence. In that way, people obtain their citizenship on an equal basis. The household system shapes the relationship between society and the state, and this is peculiar to China. The state uses the natural blood lineage to organize a household-based society and integrates with society through the superposition of households. Therefore, despite the changes of the imperial courts, families shall not be eliminated by any riots. Rather, a new dynasty would always emerge from a family (Xu and Ye 2020).

### Methodology: Historical Politics and Field Politics

The understanding of methodology encompasses three levels: first, simple research skills, such as quantitative statistics and case interviews; second, rigorous argumentation mechanisms, such as the causal mechanism, or the formal logic of empirical induction or normative deduction; third, the fundamental understanding method derived from the research object: it originates from experience but goes beyond it. In other words, its essence lies in comprehensive judgment, which is an empirical subjective point of view. The third level is called epistemology from the perspective of the generation of knowledge; ontology from the perspective of the subject of experience; and methodology from the perspective of fundamentality. Since the twenty-first century, Chinese political science has made major advances in the first two levels, making political science academically normative. The third level is critical to allowing Chinese political science to be China’s political science. Mizoguchi Yuzo’s *China as Method* and Yang Guangbin’s *Political Science with China as Method* discuss aspects related to the third level in response to “Chineseness”. In this sense, nouns are used as verbs instead of concepts in the methodological discussions, and this shows the uniqueness or analyticity of the empirical experience as indicated by the name. It is also in this sense that the “internal shift” in Chinese political science actually refers to a “shift to China”. China is not only a descriptive concept, but also an analytical one. It reflects a new form of political science.

There are two methodological concepts regarding the “internal shift”: historical politics and field politics. The idea of historical politics was first proposed by Yao Zhongqiu (Yao 2013b), but it is Yang Guangbin and his school of “historical politics” that popularized it in academic circles. Despite a lack of consensus, the school seems to have involved the entire Chinese political science community and has been reiterated by the international academic community. In terms of analytical concepts, Yang Guangbin and his colleagues’ interpretation of the “research programs” of historical politics has become increasingly clear and comprehensive. Although the specific connotation of historical politics is not defined, its issue awareness and extension or orientation are definite. As an ontological methodology, historical politics opposes the modern Western politics based on the rational man hypothesis and with institutional studies as its core. It claims that Chinese political science should “take China as the method” and respond to “Chineseness”. In essence, historical politics targets modern China, trying to re-evaluate the Chinese and Western propositions, old and new, that occurred in modern China and the historical outlook formed therefrom, and re-construct the ontology, epistemology, and methodology of Chinese political studies (Yang 2019a). A new or worldwide “yardstick” or “standard” for political knowledge is therefore created, which makes historical politics a political discourse on legality (Yang 2019b). From the perspective of academic argument, it emphasizes the need to return to or face the context of historical experience (epistemology), methodological temporality, and ontological structural relationalism, refines concepts and knowledge, and makes a summary of good governance (Yang 2020a, b, c). Historical politics has three major agendas: one, to fully describe the history of the evolution of China’s state form, reveal the origins of the contemporary state form, grasp the trend of political evolution, and explore ways to improve politics; two, to construct general theories about politics and the state through historical comparative political analysis; three, to reveal the process of political interaction between China and other countries, develop world politics history, examine the evolution history of the world political system, and explore what China can do to improve the world system (Yao 2019). These three agendas involve multiple levels of analysis from macro-standards to meso-institutions and even micro-experiences and can even be seen as a holistic political study. They are also the main force behind the internal shift in Chinese political science.

Field politics, with Professor Xu Yong of Central China Normal University at the helm, deals with the theorization of rural studies. In recent years, Professor Xu has proposed middle-range theories of field politics, including the “household system” (Xu 2013, 2016; Xu and Ye 2020), “rights from ancestors” (Xu 2018), and “China in relations” (Xu 2020a, b), which have attracted much attention in the academic world. However, there are not many methodological papers or research programs on field politics and the topics of academic lectures and roundtable forums are sporadic. Hence, Professor Xu’s methodological interpretation and knowledge construction are not very clear. In addition, from the perspective of argumentation, as to whether the “household system” and “rights from ancestors” are features of contemporary China’s social structure or a Chinese political attribute that runs through ancient and modern times is not properly supported by the relevant arguments, empirical materials, and theoretical citations; and the expression of “relations” or the “superposition of relations” is too general—its analyticity needs to be expressed more clearly.

The commonality between historical politics and field politics is that they both try to construct a way of understanding Chinese politics by returning to “Chineseness” or the meta-question of “what is China”. However, they focus on different aspects in their research orientation. Historical politics underlines “historical context” and historical resources, and experiences and concepts are placed in the scope of historical investigation. On the other hand, field politics emphasizes the induction of empirical observation (the structure of rural and agricultural societies) and involves less analysis of conceptual texts. It should be noted that although both, historical politics and field politics emphasize comparative research, they take it as their responsibility to put forth Chineseness during the research process, thus highlighting the value of focused Chinese political science or Chinese political research in the theoretical form and obscuring the transcendental dimension of comparative political science. In other words, Chinese political science exists in both, a broad sense and a narrow one. In a broad sense, it is also part of “international” social science, including and transcending the individual political knowledge forms of various regional communities and sharing a set of formal propositions for political research. Too much emphasis on “Chineseness” will detract from the explanatory power of Chinese political science as part of the “international” social science, causing the positive and open “internal shift” to be reduced to the conservative and closed “turning inward” and a shift from the “Western determinism” to the “Chinese decision”.

## “China” as Analytical Concept

In the context of Chinese political science as something that was imported since modern times, the emergence of institutional concepts in Chinese political science in recent years shows that Chinese political science is experiencing an “internal shift” that is different from the previous total Westernization. Chinese political scientists are trying to find a theoretical explanation of China’s political development from the internal context of the country and provide a knowledge argument for its modern state building. Methodologically, it means taking “China” as an analytical concept of Chinese politics.

Undoubtedly, political studies in any country as a conceptual reflection of the regional life practice, must be local. This is a common sense of epistemology. However, it is not easy for developing modern countries. This is because the linear evolution ideology of the modern international order objectively creates a split between “advanced and backward” and “civilized and barbaric”. For those countries, modernization means giving up their own traditions (backwardness and barbarism) and embracing the modern Western world. Therefore, the internal shift in Chinese political science means a reflection on and reaction against Western political theories. “China-centered” requires not only a description of China, but also an extension to the analytical meaning of it: “Why is this in China and not in other countries?”, or, “Why does China operate this way and not that way?” In other words, using “China” as an analytical concept is a response to the question of “Chineseness” or “what is China”. It is on that dimension that the institutional concept of Chinese political science has gained its positive significance. In the sense of middle-range theory, it tries to name the specific image of “China” and explore its operating mechanism. So, why is “China” an analytical concept? How does “China” become an analytical concept?

**(1) Analytical concept and its meaning.** As a way of thinking and expression, analysis is relative to description. Specifically, analysis is logical deduction and reasoning under clear and definite conceptual frameworks and focuses on causality, while description means depicting and narrating only according to some external clues (such as structure or context). Analysis, on the one hand, means that the existing concepts are clear and specific, so there will be no disagreement. On the other hand, it means that things are understood as the evolution and actions of several core variables. It is the differences in core factors that determine the differences in the nature and type of things, and analysis can reproduce the causal logical relationship between core variables through formal logic. One can deduce that if description is made from the natural perspective of structure and evolution, then analysis leads research to the actor behind the structure and the action mechanism. In this way, macroscopic research is conducted from a micro and subtle perspective. Additionally, it brings the specific and trivial micro-research into the macro vision, so that it does not neglect the overall picture. A typical expression of analytical concepts is middle-range theory and its concepts, although middle-range theory seems to be the product of a certain research technique: First, from the perspective of naming, it is the outcome selected using comparative research and its unit and lies somewhere between the macro and the micro. It is believed that if the research object is too big, it will risk hollowness, and if it is too small, it will risk narrowness. Only the middle-range theory is an appropriate option and can avoid those risks (for example, the continuous emergence of exceptional experiences make the conclusion arbitrary, and deficiency is taken as the information is not available in total). Therefore, it is a reaction against the probabilistic macro-analysis of “structural functionalism”. Second, from the perspective of a working mechanism, it focuses on the analysis and interpretation of causal logic, separates explanatory variables from the structure, and discusses changes in their causal logic. The boundary of a middle-range concept is clear, its interpretation is restrained, and it can provide new discoveries. Due to this, the literature review of social science research always takes the knowledge evolution of analytical concepts, especially the middle-range theory, as clues, hoping that new research can expand the existing knowledge chain, realize academic clustering and add value academically. Third, the premise of middle-range or analytical concepts is a comparison or classification. Classification identifies research objects and issue awareness, forming the structure of typified attention and the action mechanism behind it. Sometimes similar structures do not necessarily lead to similar results. It is decided by the internal mechanism.

Therefore, the analyticity of middle-range theory depends on the appropriate choice of unit for classification and comparison. In fact, that choice also determines whether it is macroscopic or mesoscopic. In the field of Chinese political research, “China” seems to be a large and general concept, with unclear boundaries and indefinite connotations that are yet to be confirmed; however, in country-specific comparative political science, “China” is an independent unit for comparison, and Chinese political science has been focusing on comparative studies of China and the United States, Europe, and even the West (China and the world) in modern times. Therefore, the use of “China” as an analytical concept is based on the difference between China and the West or on the dimension of comparative research. That is also the premise of the proposition of “taking China as the object and the method” in recent years.

**(2) China and its boundaries as the “object of comparative analysis”.** Nouns have natural attributes for classification and comparison. Taking “China” as an analytical concept is a comparative research method that uses nouns as analytical verbs. In the history of Western political science, there are two examples of similar nouns used as analytical verbs. Example 1: German political science or the proposition of “political science is state science” does not simply mean that political science uses the “state” as an object for empirical research, but that German political science is different from the previous political analyses of Britain and France. The difference is that in German political science, the “state (rationality/reason)” is placed above the “individual”. After concluding the “social contract theory” of the Age of Enlightenment, British political science has focused on the form of government and the operation of institutions, while German political science has put “the existence of the state” in the first place. It is in that sense that “Germany” has become an analytical concept that differs from the previous political theory. The core of German political science is “addresses to the German nation” (Fichte), which demonstrates the primacy of the state. Example 2: in American political science, “America” is also an analytical concept, because the core of American political science is neither state theory nor government theory, but institutional theories in the sense of public policy. For instance, the core issues of behavioralist and rational selectionist theories are about the political party system and the election voting behavior. In *Our Enemies and US: America’s Rivalries and the Making of Political Science* (Oren 2004), a monograph about the history of American political science, the author examines how American political science has changed along with the foreign policy (relationship between the country and its enemies). Oren declares that American political science is not a scientific and universal theory of knowledge: it is rather an ideology filled with American interests. Therefore, regional country names are used as analytical verbs because of the different forms and historical tasks of those countries, which results in differences in the focus of political knowledge. In other words, the reason why “Germany” in German political science and “America” in American political science have become analytical concepts lies in their different state forms and theoretical tasks.

Then, how is Chinese political science, which is a subordinate concept of modern political science, different from German political science and American political science? The answer probably lies in two aspects. One, as a modern state construction after Germany and America, Chinese political science reflects some universal constructions of modern states, such as the position of civil rights and democracy in the political system; two, as a political community with a long history and mature experience, Chinese political science has an internal structure and mechanism with historical continuity. “Discovering universality in China” and “discovering China in universality” are two sides of the same body, and the lack of either side would cause an imbalance.

**(3) “China” and its supporting middle-range concept group.** From the level of Chinese political research rather than comparative political science, “China” should be refined to a more specific middle-range concept when it is used as an analytical concept. Although it implies the perspective of comparative political science, it mainly presents Chinese political studies. Otherwise, it will become something too voluminous and irrelevant. In other words, considering “China” as an analytical concept requires refinement of “China” into a specific middle-range concept. In the comparative sense, a middle-range concept means that on the one hand, it leads research to the structure or traits of the research object, and on the other hand, it leads structure to actors and cultural concepts, finally forming a set of interpretation mechanisms. Specifically, the comparability of analytical concepts enables them to transcend specific objects and experiences to extract superordinate concepts, thereby realizing theoretical appreciation and refreshing knowledge. The objectivity of analytical concepts enables readers to quickly assign a name to the research object based on its traits and characteristics. In this sense, “rights from ancestors”, the “household system”, and the “superposition of relations”, just like Fei Xiaotong’s “differential mode of association”, are all theoretical expressions of Chineseness. It needs to be pointed out that although there has been no mention of “field politics” in the sense of middle-range theory, the spontaneous “internal turn” that began in the late 1990s has led to the establishment of a number of institutional concepts, such as the “pressure system”, the “(political) tournament system”, and the “consultative democracy and consensus democracy” model. From this perspective, the institutional concepts discussed above are all middle-range studies of a certain aspect of “China”.

As mentioned above, the “China” school in political science based on middle-range theory has been formed from historical politics and field politics. Their issue awareness comes from the question of “What is China” (Chineseness), and they regard Chinese history and field experience as the source of discovering and understanding China. Yang Guangbin believes that Chinese political science should “take China as the method” to respond to “Chineseness”, which is “ontology”, “epistemology”, and “methodology”, and makes it clear that Chinese political science is not a normative theory but a theory of social science. Xu Yong has two formal logics: one is comparison, in which China is to be discovered; the other is the historical repetition of Chinese social and political phenomena. Based on fieldwork, Xu came up with theoretical concepts, such as the “household system”, “rights from ancestors”, and “China in relations”. The commonality between historical politics and field politics is that they both regard “China” as an analytical concept instead of a research field. The difference is that historical politics has already developed an ambitious program, yet refinement and accumulation are still needed in terms of middle-range theory; and field politics has already yielded great results in middle-range theory or Chinese traits, but the program still needs to be improved.

**(4) The temporal and spatial orientation of “China” means that China is facing the world rather than sticking to its own land and embracing modernity rather than restoring tradition.** The internal shift in Chinese political science, which sees “China” as an analytical concept, does not mean that Chinese political science turns inward and becomes conservative “Chinese determinism”. Rather, the truth is that Chinese political science has a two-tier structure: it is first of all an international social science (pursuing universality and trans-regional argumentation), and then a Chinese political study. As opposed to “absolutism”, this proposition reflects not only the history of interaction between China and the West and the development of Chinese political science in the past century, but also the theory of China’s integration into the world as “China of the world”. “China of the world” breaks the dualistic thinking in which “China and the world” opposes “the world and China”. “China of the world” puts China within the scope of world structure and history, and examines its role, influence, and historical development, demonstrating the uniqueness of China and how it has advanced with the times. For a long time, we followed in the footsteps of the West and saw a single worldview about China and the West, that is, Western political forms and political theories represented universality, hence the act of total Westernization. However, the “internal turn of Chinese political science” tries to avoid this either-or mindset. China was once oppressed by absolutism, and in no way will it become an absolutist oppressor.

The discovery of universality in China means that there is universality that transcends geography in modern political forms, and there are also two different geographical patterns between China and the West. This is a pluralistic worldview of “universality and particularity”, which gathers people together as well as separates them.

This pluralistic worldview mirrors plural politics. As the knowledge argumentation and verbal expression of political life, there are German politics, American politics, Chinese politics, and perhaps Islamic politics in the future. Within Chinese political science, there are also many political discourses that have different research origins, analysis paths, focuses, and outlooks for the future. However, as they are all born out of China-centered research, which “takes China as the object and the method”, they are simply placed under Chinese political science in general. “Universality and particularity” do not head toward relativism and nihilism like modernity studies. Instead, it presents a pluralist expression under the meta-topic of political community.

**(5) The historical limitation or the dialectical unity of subject and object regarding “China” as an analysis object and analytical concept.** The dialectical unity of subject and object determines that “China” becomes the analysis object of political research. The integrity or perfection of the state and its system is of utmost significance (“a mature or fixed state or system”) with respect to the object. The current internal shift in Chinese political science, which occurs as China is rising in the twenty-first century, is not the same as the “Chinese-centered cultural declaration” of the 1930s, when the country was experiencing a devastating political situation. With regard to subject, “your eyes shape what you see” and “your ears determine what you hear”: the subject’s knowledge structure and experience prime the eyes and ears for observation. The discovery of “China” in Chinese political science is a combination of the above two: first, the rise of “China” and the changes in the international environment determine the making of “China” as a research object; second, through their comprehension and experience of and reflection on Chinese and Western political theories, political scientists have paid attention to localization in terms of history and theory. The “re-discovery/re-description of China” promoted by empirical research methods, in particular, has made more Chinese phenomena the subject of empirical research. Therefore, it is a dialectical unity of the internal turn of Chinese political science that “China” becomes an analyzable object and that “China” is analyzed from the perspective of social science.

Using "China" as an analytical concept, or using nouns as analytical verbs, has two meanings: universality and particularity, which are dialectically unified. Nouns are local and they reflect regional culture, yet the theory behind nouns is beyond the regional genus relationship. Therefore, as the analysis core of the internal turn of Chinese political science, "China" is, first of all, a proposition of comparative political science. Different regions have regional plans to deal with modernity or modern countries, and their demonstration methods conform to the formal logic of comparative political analysis, which shows universality; second, it is a Chinese proposition, and its empirical evidence and specific state come from traditional and modern fields in China. In that specific state, it is different from the experience of other regional communities. Only by understanding "China" as a dialectical unity of universality and particularity, can the internal turn of Chinese political science achieve a balance in terms of theory.

## Chinese Political Science Makes a New Beginning

Chinese political knowledge and its modern discourse have undergone a series of transformations since modern times. Traditional China had its own classifications of knowledge. In modern times, due to the encounter with the West, the classifications of traditional Chinese classics were "compiled" into the modern academic branch system of the West. Later, traditional mainstream Chinese political words and phrases were replaced by "waseikango", and the conceptual understanding of modern China was separated from the traditional and local ones at the level of discourse. Traditional discourse or local expressions were regarded as backward and despicable. Therefore, it is no exaggeration to say that right from the words used to make sentences to the worldviews attached to them, knowledge generation was totally Westernized in modern times.

The sinicization of Chinese political science, which is an imported discipline, is also the first part of its Westernization. From the political science courses of the new-style schools, to the subject of political science at Imperial University of Peking, and to the departments of political science of Peking University and other modern universities, they were largely based on Westernized courses and teaching materials. After 1978, political science was re-built and the Westernization was further confirmed and deepened in the discipline evaluation index system. Hence, we find traces of American political science in the discipline buildup of Chinese political science, ranging from concepts and contents to forms and activities. Under such a methodology, a paper written in Chinese discusses about China, yet "China" is not regarded as an independent political community where experiences are discovered, and theories produced. Chinese political science or Chinese research has become an extended vision and a sub-field (Sinology) of Western political science. Since the 1980s, in particular, Chinese political science has become an extension of the experience field of the geopolitics-based comparative political research in America. Since the 1980s, Chinese political studies have been dominated by concepts and theories like revolution and modernization, democracy and totalitarianism, governance and good governance. As a result, China has become a testing ground for the confirmation or falsification of American political science: the proposition of Chinese political science comes from America; Chinese experience is used to prove the correctness of the American theory, or the American theory is directly applied to analyze Chinese experience; and even the legitimacy of China's reform practice should be confirmed by American political theory. Most notably, the results of Chinese social science research would be recognized only if they are published in Social Science Citation Index (SSCI) journals, which are European and American academic journals with specific preferences for topics and methodologies. That is also an official indicator of discipline evaluation by the Ministry of Education of China. In short, Chinese political science over the past century has understood Western political science, especially American political science, as the world or comparative political science, and the institution and theory summarized based on the Western regional political experience as the paradigm of world politics. The absence of "China" and the theoretical imbalance between China and the West in terms of regional political experience have caused "total Westernization" in the hundred-year history of Chinese political science, where "China is absent".

To be fair, Chinese political knowledge has an instinct for localization in the face of the Western knowledge system based on modern academic branches. Kang Youwei's "Reform under the cover of antiquity", Zhang Zhidong's "Chinese essence and Western utility" and Yan Fu's translation into Chinese can all be regarded as the spontaneous "resistance" of traditional Chinese scholars, which is also demonstrated by the tragic deaths of Wang Guowei and Liang Ji (Liang Shuming's father) in the twentieth century. In the 1930s, "Ten Professors' Declaration of China-centered Cultural Buildup" showed, culturally and emotionally, greater concerns about localization when the country was damaged in the Anti-Japanese War. In the late 1990s, the split between the new left and the intellectual circle with the knowledge about Western modernity showed concerns about localization between the two divisive orientations of state construction—the modern state construction and the modern state deconstruction. Afterward, Deng Zhenglai, Jing Yuejin, and other social scientists launched the *Chinese Social Sciences Quarterly* (later renamed as the *Chinese Social Science Journal*) to demonstrate the independence of Chinese social science, and Deng Zhenglai published *Where is Chinese Law Going*. Several social science researchers on law, political science, and sociology explored and reflected on the "state-society" (civil society) theory to "rediscover China", calling for the localization of Chinese social science. Since the twenty-first century, political scientists like Guo Sujian have once again asked: "where is China's political discipline heading" (Guo 2018), which means that the development trend of Chinese political science is increasingly moving away from the "form" of discipline formation toward the "content" of "what is China".

In recent years, the formation and rise of the institutional concept of Chinese political science has led to this changing trend. On the one hand, it means that Western political theories cannot explain the experience of "China's rise", or understand the logic of China developing as a community from tradition to revolution to development and even to governance, leaving a blank space in comparative political science (Jing 2019). On the other hand, the experience of China's political development reflects the inherent predicament of European and American countries as declared by Western critical theories and the analyzability of the Chinese experience caused by it. The internal turn of Chinese political science is taking place in the context of the political interplay between China and the world. Also, from the perspective of specialized knowledge production, the "internal shift" mainly stems from the long-term accumulation of Chinese political science knowledge (especially state-building theories including Marx's critique, Western modernization and modernity) and rational thinking about China's rise and the transformation of the world situation. Scholars who have made outstanding research in the field of this "internal shift" have a composite knowledge structure of "Marxism", "modern West", and "traditional China", although they may be inferior to their predecessors in certain areas: they may read fewer original Western works and are less familiar with text than the new leftist scholars who have studied abroad; and they may not understand traditional Chinese political science as profoundly as Zhang Zhidong and Kang Youwei. However, their apprehension about the value, their reflection, criticism, and refusal to blindly believe in one particular ideology, their constructive mindset of "history–society–culture" based on "understanding and sympathy", and more importantly, their observation and comparison of contemporary political practice in China and the West, jointly contribute to this internal shift. As far as the particular field of China is concerned, their views are different from those of the European and American leftists, as well as pure traditional researchers or reactionists.

Although the institutional concept of the internal shift in Chinese political science may not have been agreed upon by the academic community, and the internal shift has not even emerged in some areas, it has at least succeeded in achieving the first step from vision to experience. As an empirical research object, it outlines the knowledge domain, issue context, and interpretation mechanism of Chinese political science. As a target "to be revised", institutional concepts can be supplemented, revised, added, deleted, or even replaced in reflection and questioning and finally bear fruitful theoretical fruit. It is in this sense that Chinese political science, after a long period of formal construction, has begun to make a real new beginning.

I find it appropriate to conclude this paper with the last paragraph of another article: "Chinese political research has reached a tipping point. It is like the spring silkworm's cocoon: after getting rid of the old form, it will take on a more beautiful appearance. If we can make a breakthrough in the understanding of the discipline and China, we will bring forth the universal value of Chinese political science and the history of it, providing the world with new concepts of interpretation and research paradigms." (Wang 2021).

## Footnotes

1 Comparative political science is used instead of comparative research/comparative politics because in the context of Chinese political science, "comparative political science" is often used to understand and parallel "comparative research/comparative politics" in American political science. The discrepancy in the wording may cause a departure from the original meaning of "comparative research/comparative politics", but the term "comparative political science" has been retained to reflect what the Chinese political circle really thinks. As far as this paper is concerned, Chinese political science constructs its own significance in the original dimension of comparative research.
